# Alpha-Helical Destabilization of the Bcl-2-BH4-Domain Peptide Abolishes Its Ability to Inhibit the IP_3_ Receptor

**DOI:** 10.1371/journal.pone.0073386

**Published:** 2013-08-30

**Authors:** Giovanni Monaco, Elke Decrock, Koen Nuyts, Larry E. Wagner II, Tomas Luyten, Sergei V. Strelkov, Ludwig Missiaen, Wim M. De Borggraeve, Luc Leybaert, David I. Yule, Humbert De Smedt, Jan B. Parys, Geert Bultynck

**Affiliations:** 1 Laboratory of Molecular and Cellular Signaling, Department of Cellular and Molecular Medicine, Leuven, Belgium; 2 Department of Basic Medical Sciences, Physiology Group, Faculty of Medicine and Health Sciences, Ghent University, Ghent, Belgium; 3 Section of Molecular Design and Synthesis, Department of Chemistry, Heverlee, Belgium; 4 Department of Pharmacology & Physiology, School of Medicine and Dentistry, University of Rochester Medical Center, Rochester, New York, United States of America; 5 Laboratory for Biocrystallography, Department of Pharmaceutical and Pharmacological Sciences, Leuven, Belgium; Indiana University School of Medicine, United States of America

## Abstract

The anti-apoptotic Bcl-2 protein is the founding member and namesake of the Bcl-2-protein family. It has recently been demonstrated that Bcl-2, apart from its anti-apoptotic role at mitochondrial membranes, can also directly interact with the inositol 1,4,5-trisphosphate receptor (IP_3_R), the primary Ca^2+^-release channel in the endoplasmic reticulum (ER). Bcl-2 can thereby reduce pro-apoptotic IP_3_R-mediated Ca^2+^ release from the ER. Moreover, the Bcl-2 homology domain 4 (Bcl-2-BH4) has been identified as essential and sufficient for this IP_3_R-mediated anti-apoptotic activity. In the present study, we investigated whether the reported inhibitory effect of a Bcl-2-BH4 peptide on the IP _3_R1 was related to the distinctive α-helical conformation of the BH4 domain peptide. We therefore designed a peptide with two glycine “hinges” replacing residues I14 and V15, of the wild-type Bcl-2-BH4 domain (Bcl-2-BH4-IV/GG). By comparing the structural and functional properties of the Bcl-2-BH4-IV/GG peptide with its native counterpart, we found that the variant contained reduced α-helicity, neither bound nor inhibited the IP _3_R1 channel, and in turn lost its anti-apoptotic effect. Similar results were obtained with other substitutions in Bcl-2-BH4 that destabilized the α-helix with concomitant loss of IP_3_R inhibition. These results provide new insights for the further development of Bcl-2-BH4-derived peptides as specific inhibitors of the IP_3_R with significant pharmacological implications.

## Introduction

Intracellular Ca^2+^ homeostasis requires a tight cross-talk between the endoplasmic reticulum (ER) and the mitochondria. Although mitochondria need basal levels of Ca^2+^ to sustain cellular bioenergetics demands, mitochondrial Ca^2+^ overload leads to the onset of mitochondrial outer membrane permeabilization (MOMP) and downstream apoptosis activation [1,2]. Anti-apoptotic Bcl-2 family members have a dual role in MOMP prevention: 1) they antagonize the pore-forming activity of their pro-apoptotic relatives, BAX and BAK, on mitochondria and 2) they fine-tune the ER-mitochondria interplay towards pro-survival or anti-apoptotic Ca^2+^ signals [3–5]. There is now increasing evidence that Bcl-2, localized at the ER membranes, controls the ER Ca^2+^ content and Ca^2+^ release. It was suggested that Bcl-2 could exert its protective function by decreasing the luminal Ca^2+^ content *via* an interaction with the sarco/endoplasmic-reticulum Ca^2+^-ATPase (SERCA) [6,7] or more generally by increasing the passive leak of Ca^2+^ across the ER membrane [8–10]. In addition, Eckenrode et al. [11] proposed a direct interaction of anti-apoptotic proteins (Bcl-2, Bcl-Xl and Mcl-1) with the C-terminus of inositol 1,4,5-trisphosphate receptors (IP _3_Rs), increasing the activity of these ER channels and therefore decreasing the steady-state [Ca^2+^]_ER_. Herein we further established a role for the last transmembrane domain of the IP_3_R for Bcl-2/Bcl-Xl interaction [12]. On the other hand, Distelhorst and collaborators as well as our own group have demonstrated that Bcl-2, by interacting with the central, modulatory region of the IP_3_R, inhibited pro-apoptotic Ca^2+^ signals from the ER without affecting steady-state Ca^2+^ concentration in the ER ([Ca^2+^]_ER_) [12,13]. As this Bcl-2-binding site is largely conserved between the different IP_3_R isoforms [12], the latter inhibitory mechanism is considered a common denominator among IP _3_R1, IP _3_R2 and IP _3_R3 channels.

Notably, the apparently divergent molecular mechanisms described above converge into a reduction of the pro-apoptotic Ca^2+^ transfer from the ER to mitochondria. However, distinct Ca^2+^ signals may have opposite outcomes, as Ca^2+^ oscillations may promote cell survival by boosting mitochondrial bioenergetics, while Ca^2+^ overload may result in cell death by triggering mitochondrial outer membrane permeabilization [14]. Consequently, Bcl-2 proteins may modulate both Ca^2+^-signaling modes with differential regulation by distinct Bcl-2-family members or distinct protein domains [15]. Moreover, it is clear that these anti-apoptotic proteins not only play an important role in controlling Ca^2+^ signaling in healthy cells, but may also contribute to dysfunctional Ca^2+^ signaling in diseases, like cancer [16].

Our previous data identified the BH4 domain as an essential and sufficient component of Bcl-2 responsible for the direct inhibition of IP_3_-induced Ca^2+^ release (IICR) and apoptosis [12,13,17]. Furthermore, the BH4 domain is essential for many anti-apoptotic members of the Bcl-2 family (like Bcl-2 and Bcl-Xl) since its deletion abrogates their anti-apoptotic activity [18–20]. Remarkably, the isolated Bcl-2-BH4 domain was sufficient to protect against Ca^2+^-mediated apoptosis by selectively acting on the IP _3_Rs [12,17], whereas the very similar Bcl-Xl-BH4 domain did not show such IP_3_R-dependent protective activity [12,15].

**Figure 1:**
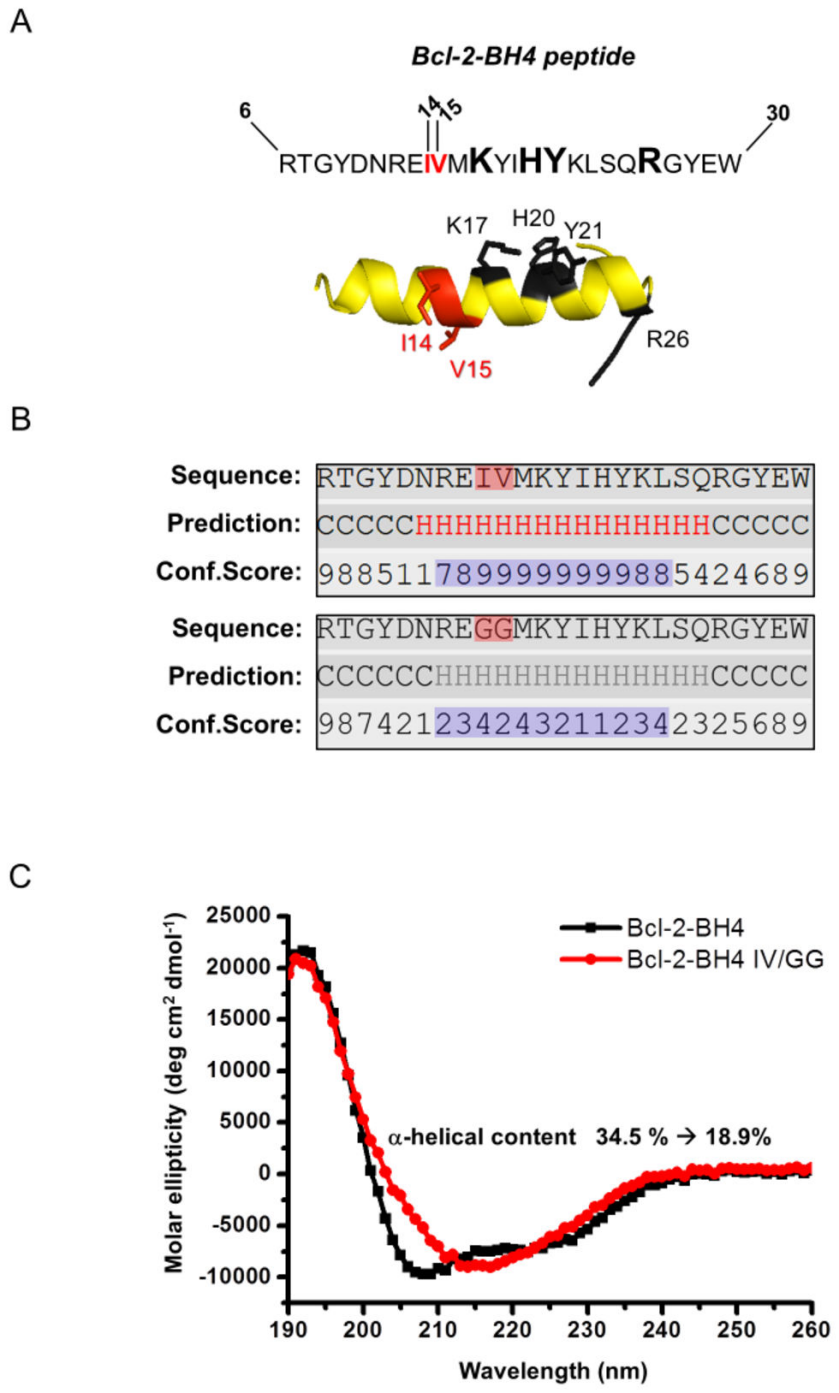
A Bcl-2-BH4 double-glycine variant (I14G/V15G) has a decreased **α-helical content**. (A) Primary structure of the Bcl-2-BH4 peptide. The key residues involved in the regulation of IP _3_Rs are depicted in black/bold. The residues considered for the glycine substitution (I14 and V15) are depicted in red. The α-helical structure underneath represents the best predictive model obtained from I-TASSER web server and drawn using Pymol. The labels for the key peptide residues follow the same color code as in the primary structure. (B) Predicted-secondary structure assignments for the isolated BH4 domain of Bcl-2 (upper panel) and for its IV/GG counterpart (lower panel). Each panel shows the amino acid sequence, the secondary-structure predictions (H = α-helix; C = random coil) and the level of confidence of the predictions (confidence scores from 0 to 9). Residues 14 and 15 of the BH4 domain are highlighted by a semi-transparent red square. (C) CD spectra of synthetic Bcl-2-BH4 (black line) and Bcl-2-BH4 IV/GG peptides (red line). The ellipticity is calculated per mole of amino-acid residue. Bcl-2-BH4-IV/GG peptide lost the native α-helical conformation to adopt a more β-sheet-like structure (210 nm-ellipticity minimum). For the percentages of the other secondary structure features see Table S1.

The functional BH4 domain in the native N-terminal domain of Bcl-2, comprises a stretch of 20 amino acids (a.a. 10 to 30) organized in an α-helical structure (α1) [21,22]. As we previously showed, some residues of the Bcl-2-BH4 domain (K17, H20, Y21 and R26, Figure 1A) coordinate the inhibitory function of the Bcl-2-BH4 peptide on the IP _3_Rs [12]. These residues are highly surface-accessible in the native Bcl-2 protein and proximal in the secondary structure-backbone [13]. Hence, we hypothesized that the Bcl-2-BH4 peptide may need a stable α-helical structure for inhibiting the IP_3_R-channel activity. To test our hypothesis, we selected, by *in silico* analysis, a peptide modification that is predicted to affect BH4-α-helical stability. More specifically, we used a modified version of the Bcl-2-BH4 peptide that carries a change in two hydrophobic residues, which are part of the N-terminal α -helix of the native Bcl-2 but display a poor surface accessibility in the full-length protein [13,22,23]. In this peptide, residues I14 and V15 have been replaced with two glycines to introduce high flexibility in the structure and destabilize helical conformation of the peptide. We applied this modified peptide in a series of functional experiments, addressing its ability to bind IP _3_R1, inhibit single-channel activity, curb IICR and protect against Ca^2+^-dependent apoptosis. We further examined the relevance of the α-helical domain organization of the BH4 domain by introducing other mutations that reduce the α-helical content.

## Materials and Methods

### Peptides and plasmids

The following peptides, obtained from Thermo Electron, Germany, were used: Bcl-2-BH4: *RtgydnreivmKyihyklsqrgyew*; Bcl-2-BH4 SCR: *WYEKQRSLHGIMYYVIEDRNTKGYR*; Bcl-2-BH4 IV/GG: *RtgydnreGGmKyihyklsqrgyew*; BH4-Bcl-2 II/GG: *RTGYDNREGVMKYGHYKLSQRGYEW*; BH4-Bcl-2 VIL/GGG: *RTGYDNREIGMKYGHYKGSQRGYEW*. The amino acids replaced by glycines are, respectively, I14-V15, I14-I19 and V 15-I19-L23. All peptides were more than 80% pure and their identity was confirmed *via* mass spectrometry (MS). The pGEX-6p2 construct (Amersham Biosciences, GE healthcare) encoding amino acids 923-1581 of mouse IP _3_R1 Domain 3 was obtained as previously described [12].

### Cell culture and transfections

Mouse Embryonic Fibroblasts (MEF cells) [24] were cultured at 37°C in a 9% -CO_2_ incubator in DMEM/Ham’s F12 medium (1:1) (Invitrogen, Belgium) supplemented with 10% fetal calf serum (Sigma-Aldrich), 3.8 mM L-glutamine (Glutamax, Invitrogen), 85 IU/ml penicillin and 85 µg/ml streptomycin (Invitrogen). Rat C6 glioma cells [25,26] were cultured in DMEM/Ham’s F12 medium (1:1), containing 10% fetal calf serum, 100 IU/ml penicillin, 100 µg/ml streptomycin, 2.5 µg/ml fungizone and 2 mM L-glutamine at 37°C and 5% CO_2_. DT40-3KO cells, stably expressing rIP _3_R1 [27], were cultured at 39°C in a 5% CO_2_ incubator in RPMi (Invitrogen) supplemented with 2.05 mM L-glutamine, 10% fetal calf serum, 1% chicken serum, 100 IU/ml penicillin, and 100 µg/ml streptomycin (Invitrogen).

### Peptide stability and secondary-structure predictions

To calculate the change in pseudo-thermodynamic stability induced by the GG substitutions, we used Eris, an automated estimator of free-energy-change variations (ΔΔG), with unbiased force field, side-chain packing and backbone relaxation algorithms [28]. First, we extrapolated the structure of the Bcl-2-BH4 peptide from the PDB file corresponding to the NMR structure of Bcl-2 protein (PDB: 1G5M [21]) and submitted it successively to the prediction server. The ΔΔG values reported in this study are indicated in kcal/mol. Additionally, the I-TASSER v 2.1 webserver [29] was used to predict the secondary structure of the different BH4 peptides starting from their amino-acid sequence. I-TASSER builds protein models using iterative assembling procedures and multiple threading alignments from template structures libraries. An estimate of accuracy of the predictions is given by the confidence score. In the case of Bcl-2-BH4 peptide, the most accurate I-TASSER model was downloaded as PDB file and imported in PyMOL, a molecular graphics software (http://www.pymol.org).

### CD spectroscopy

CD spectra (Jasco J-810 circular dichroism spectropolarimeter, USA) were recorded at 1 nm intervals over the 190-260 nm wavelength range using 1 mm path length quartz cells (Hellma, Germany). Ten scans were performed. The experiments were run at 20 nm/min and the temperature was fixed at 298^°^ K (25 °C). The secondary structure of the peptide (50 µM) was determined in 97.5% 2,2,2-trifluoroethanol (TFE) and 2.5% methanol. The ellipticity is calculated per mole of amino-acid residue. To estimate the secondary structure of the peptide in terms of α-helical content, an analysis of the relevant CD spectra was carried out using the CDPro software (http://lamar.colostate.edu/~sreeram/CDPro/main.html) developed by Woody and coworkers [30]. The basis set 10 of the CDPro software was used [31]. The analysis was performed using the CONTIN/LL [31], a self-consistent method with an incorporated variable selection.

### Preparation of GST-fusion proteins

BL21(DE3) *Escherichia coli* cells were transformed with pGEX-6p2 constructs containing cDNAs of IP _3_R1 Domain 3 (GST-Dom3 IP _3_R1: aa 923-1581). The expressed protein was purified as previously described [32]. GST-fused-IP _3_R1 Domain 3 was affinity purified and dialyzed against standard phosphate-buffered saline (PBS) without added Ca^2+^ or Mg^2+^ (2.67 mM KCl, 1.47 mM KH_2_PO_4_, 137.93 mM NaCl, 8.06 mM Na_2_HPO_4_; Invitrogen) using Slide-A-Lyzer with a cut-off of 3 kDa (Thermo, Fisher Scientific, USA). After dialysis, the concentration of the purified GST-fusion protein was determined using BCA Protein-Assay Reagent (Thermo, Fisher Scientific), and the quality and integrity were examined by SDS-PAGE and GelCode^TM^ (Thermo, Fisher Scientific) blue stain reagent prior to GST-pull downs.

### GST-pull-down assays

Equal amounts (30 µg) of the intact full-length GST-Dom3 IP _3_R1 or parental GST (control) were incubated in Interaction Buffer (50 mM Tris-HCl, 300 mM NaCl, 1 mM EDTA, 1% NP-40, 0.5% sodium deoxycholate, 0.5% bovine serum albumin (BSA) and protease inhibitor cocktail, pH 7.0) with 30 µg of different BH4 domains (Bcl-2-BH4, Bcl-2-BH4 IV/GG) and immobilized on glutathione-Sepharose 4B beads (GE Healthcare, Europe GmbH, Belgium) *via* rotation in a head-over-head rotator for 2 h at 4°C. The beads were washed 4 times with modified Interaction Buffer (150 mM NaCl instead of 300 mM NaCl, without BSA) and complexed GST-fusion proteins were eluted by incubating the beads with 40 µl LDS® (Invitrogen) for 3 min at 95°C and collected after centrifuging at 500 g for 5 min. Eluates (10 µl) were subjected to SDS-PAGE.

### SDS-PAGE

Sample protein concentrations were determined by Bradford assay (Sigma-Aldrich) using BSA as standard. Proteins (10-20 µg) were separated by NuPAGE 4–12% Bis/Tris SDS-polyacrylamide gels using MES/SDS-running buffer (Invitrogen). The gels were stained with GelCode^TM^ blue stain reagent following the manufacturer recommendations. The resulting bands were quantified by using ImageJ software (rsbweb.nih.gov/ij/).

### Unidirectional ^45^Ca^2+^-flux assay

^45^Ca^2+^-unidirectional flux experiments were performed as previously described [12]. Twelve-well clusters containing MEF cells were fixed on a thermostated plate at 30^o^C on a mechanical shaker. The culture medium was aspirated, and the cells were permeabilized by incubating them for 10 min in a solution containing 120 mM KCl, 30 mM imidazole-HCl (pH 6.8), 2 mM MgCl_2_, 1 mM ATP, 1 mM EGTA and 20 µg/ml saponin. The non-mitochondrial Ca^2+^ stores were then loaded for 45 min in 120 mM KCl, 30 mM imidazole-HCl (pH 6.8), 5 mM MgCl_2_, 5 mM ATP, 0.44 mM EGTA, 10 mM NaN_3_ to prevent mitochondrial Ca^2+^ uptake, and 150 nM free ^45^Ca^2+^ (28 µCi/ml). Then, 1 ml of efflux medium containing 120 mM KCl, 30 mM imidazole-HCl (pH 6.8) and 1 mM EGTA was added and replaced every 2 min. IP_3_ (3 µM) was added for 2 min after 10 min of efflux. At the end of the experiment, all ^45^Ca^2+^ remaining in the stores was released by incubation with 1 ml of a 2% (w/v) sodium dodecyl sulfate solution for 30 min. Ca^2+^ release was plotted as fractional loss (% / 2 min) as a function of time as previously described [12]. The effect of the BH4-domain peptides on IICR was tested by pre-incubating the peptides for 4 min before exposing the stores to IP_3_.

### Electroporation loading

*In situ* electroporation of adherent C6 cell monolayer cultures was performed as previously described [25], according to a procedure optimized for cell-death studies [33,34]. C6 cells were grown to near confluency on 13 mm- (apoptosis experiments) or 18 mm- (flash photolysis of caged IP_3_ and [Ca^2+^]_cyt_ imaging) diameter glass coverslips. Cell monolayers were washed 3 times with Hanks’ balanced salt solution buffered with HEPES (HBSS-HEPES) supplemented with D-glucose (0.81 mM MgSO_4_, 0.95 mM CaCl_2_, 137 mM NaCl, 0.18 mM Na_2_HPO_4_, 5.36 mM KCl, 0.44 mM KH_2_PO_4_, 5.55 mM D-glucose, 25 mM HEPES, pH 7.4) and subsequently 3 times with a low-conductivity electroporation buffer (4.02 mM KH_2_PO_4_, 10.8 mM K_2_HPO_4_, 1.0 mM MgCl_2_, 300 mM sorbitol, 2.0 mM HEPES, pH 7.4). They were placed 400 µm underneath a two-wire Pt Ir electrode on the microscopic stage and electroporated in the presence of a tiny amount of electroporation solution (10 µl). Electroporation was done with 50 kHz bipolar pulses applied as trains of 10 pulses of 2 ms duration each and repeated 15 times. The field strength was 100 V peak-to-peak applied over a 500 µm electrode separation distance. After electroporation, cells were thoroughly washed with HBSS-HEPES.

### Electrophysiology

Isolated DT40 nuclei were prepared by homogenization as previously described [27]. 3 µl of nuclear suspension were placed in 3 ml of bath solution which contained 140 mM KCl, 10 mM HEPES, 500 µM BAPTA, and 246 nM free Ca^2+^, pH 7.1. Nuclei were allowed to adhere to a plastic culture dish for 10 minutes prior to patching. Single IP_3_R channel potassium currents (i_k_) were measured in the on-nucleus patch clamp configuration using PClamp 9 and an Axopatch 200B amplifier (Molecular Devices, Sunnydale, California) as previously described [35]. Pipette solution contained 140 mM KCl, 10 mM HEPES, 1 µM IP_3_, 5 mM ATP, 100 µM BAPTA, and 200 nM free Ca^2+^. Effects of the BH4 peptides on channel activity were examined by including 50 µM of the appropriate peptide in the pipette solution. Traces were recorded at -100 mV, sampled at 20 kHz and filtered at 5 kHz. A minimum of 15 seconds of recordings were considered for data analyses. Pipette resistances were typically 20 MΩ and seal resistances were >5 GΩ. Single channel open probabilities (P_o_) were calculated by half-threshold crossing criteria using the event detection protocol in Clampfit 9. We assumed that the number of channels in any particular nuclear patch is represented by the maximum number of discrete stacked events observed during the experiment. Even at low P_o_, stacking events were evident [27]. Only patches with 1 apparent channel were considered for analyses.

### Flash photolysis of caged IP_3_ and [Ca^2+^]_cyt_ imaging

Changes in [Ca^2+^]_cyt_, triggered by photolytic release of IP_3_ from a caged inactive precursor (caged IP_3_; Invitrogen), were monitored according to a protocol described in detail in [34]. Briefly, C6 cells were seeded on 18 mm-diameter glass coverslips and ester-loaded for 25 min with 5 µM Fluo-3-AM (Invitrogen) in HBSS-HEPES supplemented with 1 mM probenecid (Sigma-Aldrich) and 0.01% pluronic F-127 (Invitrogen) at 37°C, followed by de-esterification over 15 min. Subsequently, cells were loaded with 100 µM Dextran Tetramethyl Rhodamine (DTR) (Invitrogen), 20 µM BH4 peptides and 50 µM caged IP_3_ (Invitrogen) using the *in situ* electroporation technique as described above. Imaging was carried out using a Nikon Eclipse TE300 inverted epifluorescence microscope equipped with a 40x oil-immersion objective (Plan Fluor, NA 1.30; Nikon) and an EM-CCD camera (QuantEM™ 512SC CCD camera, Photometrics, Tucson, AZ). The UV flash (349 nm UV DPSS laser, Explorer; Spectra-Physics, Newport, Irvine, USA) was applied at 5 different places along the electroporated area per dish. Images (1/s) were generated with software written in Microsoft Visual C^2+^6.0. Fluorescence-intensity changes in all cells in a predefined 3950 µm^2^ region were analyzed with custom-developed FluoFrames software (generated by L.L. and collaborators, Univ. Gent, Belgium). For each individual trace, we calculated the relative change of Fluo-3 fluorescence (ΔF/F). ΔF/F equals [F_t_-F_0_/F_0_], with F_0_ denoting the fluorescence before application of the UV flash and F_t_ the fluorescence at different time points after the UV flash. Subsequently, relative [Ca^2+^]_cyt_ changes were quantified as the area under the curve of the various Ca^2+^ traces. Data were normalized to the vehicle condition, which was set as 100%. A minimum of 5 dishes have been used for each condition.

### Apoptosis induction and detection

C6 glioma cells were electroporated in the presence of 100 µM DTR and 20 µM BH4 peptides. After electroporation, cells were kept in 200 µl culture medium containing 2 µM STS (Sigma-Aldrich). Six hours later, cultures were stained first with 10 µM of the CaspACE FITC-VAD-FMK ‘*In situ* Marker’ (Promega, Benelux, Leiden, The Netherlands) in HBSS-HEPES for 40 min at 37°C. After fixing the cells with 4% paraformaldehyde for 25 min at room temperature, nuclei were additionally stained for 5 min with 1 µg/ml DAPI (Sigma) in PBS supplemented with Ca^2+^ and Mg^2+^ (PBSD+). Cells were mounted with Vectashield fluorescent mounting medium (VWR International, Leuven, Belgium) on glass slides. Five images (in each culture) were taken in the electroporated area and five outside the electroporated area using a Nikon TE300 epifluorescence microscope equipped with a 10x objective (Plan APO, NA 0.45; Nikon) and a Nikon DS-Ri1 camera (Nikon, Brussels, Belgium). The number of caspase-positive cells and DNA-fragmented nuclei were counted in each image, expressed relative to the number of nuclei present and indicated as the apoptotic index (AI). AI is defined as the ratio between dead cells and the total number of cells. Small groups of apoptotic bodies were counted as remnants of a single apoptotic cell. Analyses were carried out blinded, making use of custom-developed counting software. The AI in the electroporated area was expressed relative to the AI outside the area.

### Data and statistical analysis

Data are expressed as means ± SEM, unless a typical experiment is shown (mean ± SD). Statistically significant differences were considered at P<0.05 (single symbols), P<0.01 (double symbols) and P<0.001 (triple symbols) after using a two-tailed paired Student’s *t* test (Excel Microsoft Office) or one-way ANOVA and a Bonferroni post-test using Origin7.0.

## Results

### *α*-helix destabilization by I14G/V15G substitution in the Bcl-2-BH4-domain peptide (Bcl-2-BH4 IV/GG)

To maximize the chances of obtaining a peptide with low helical propensity, we opted for replacing two adjacent amino acids in the core of the Bcl-2-BH4 sequence with glycines. Glycines are well-known helix destabilizers by introducing an excessive degree of flexibility in the helix backbone [36,37]. The following rationale was adopted to select the target amino acids for the GG substitution: we avoided the residues previously proposed to be important in the IP_3_R interaction (K17, H20, Y21 and R26) (bold in Figure 1A), but we still aimed at the core of the α-helical structure. We focused on two residues that certainly participate in forming the α-helix (I14, V15) but are buried in the native structure of the full-length Bcl-2 protein and therefore not available for possible molecular interactions [13,23] (Figure 1A, 1B upper panel). By *in silico* analysis, we first predicted the change in thermodynamic stability and secondary structure induced by a double glycine substitution in position 14 and 15. This analysis suggested that altering I14/V15 into glycines in Bcl-2-BH4 will induce a high degree of structure destabilization (ΔΔG > 8.4 kcal/mol). Accordingly, the I-TASSER webserver (http://zhanglab.ccmb.med.umich.edu/I-TASSER/) suggests that while an α-helical structure covers at least 40% of the wild-type BH4-peptide sequence (Figure 1B, upper panel), Bcl-2-BH4 IV/GG displays a substantially weakened α-helical conformation (Figure 1B, lower panel).

To validate the *in silico* findings, we performed circular dichroism (CD) spectroscopy and compared the propensity of Bcl-2-BH4 and Bcl-2-BH4 IV/GG peptides to form an α-helix in TFE. This solvent is routinely used as a medium for determining the α-helical propensity of small polypeptides by CD spectroscopy [38]. The far-UV CD spectrum of Bcl-2-BH4 demonstrated the presence of a stable α-helical structure, by showing the typical two spectral minima at 208 and 222 nm. A different pattern was observed for the Bcl-2-BH4 IV/GG mutant, which displayed a singular minimum of ellipticity detectable at about 215 nm (Figure 1C). Therefore, the mutant appears to have less propensity to form an α-helix, as compared to the wild-type BH4 domain, and rather displays a β-sheet-like structure [39]. Deconvolution of the CD traces provided us with an α-helical content of 34.5% and 18.9% for Bcl-2-BH4 and Bcl-2-BH4 IV/GG, respectively (for the percentages of the other secondary structure features see table S1). This initial analysis indicated that amino acids I14 and V15 are important to stabilize the native α-helical conformation of the Bcl-2 BH4 peptide.

### In contrast to Bcl-2-BH4, Bcl-2-BH4 IV/GG fails to bind IP_3_R and inhibit its single-channel activity

**Figure 2:**
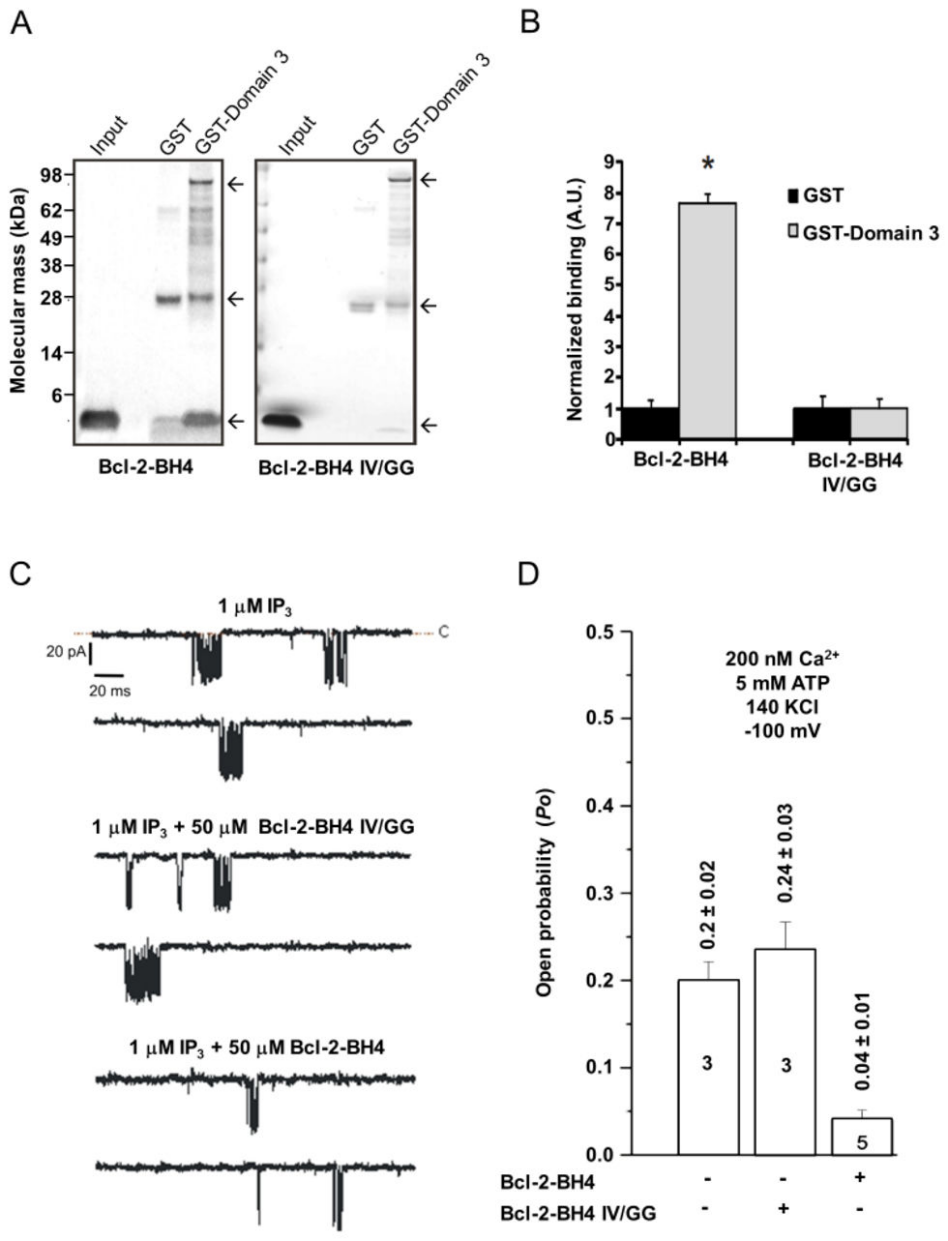
IV/GG substitution in BH4-Bcl-2 abolishes the direct interaction with IP _3_R1-Domain 3 and the resulting inhibition of IP_3_R-channel activity. (A–B) Pull-down assays of BH4 peptides with either purified GST-Domain 3 or GST alone. (A) Specific interactions between Bcl-2-BH4 or Bcl-2-BH4 IV/GG-peptides (30 µg) and the GST proteins (30 µg) detected by total protein staining (GelCode^®^ Blue Stain Reagent) of SDS-PAGE runs. The arrows indicate the bands for GST-domain-3 (upper arrow), GST (middle arrow) and BH4-domain peptides (lower arrow). (B) Bands corresponding to BH4-domain peptides were quantified using ImageJ software. Values were normalized relatively to the binding to GST and corrected for the amount of GST-fusion proteins. The results of at least 4 independent experiments are plotted as means ± SEM. * indicates a statistically significant difference from the GST control. (C) Representative single-channel recordings evoked by low [IP_3_] (1 µM) at 200 nM Ca^2+^ and 5 mM ATP, in the presence or absence of the BH4 peptides. (D) Histogram depicting the open probability (Po) ± SD for the IP _3_R1 under the previously described conditions. Within every bar is indicated the total number of recordings per each condition. The Po for IP _3_R1 was ^~^5 fold lower when exposed to the Bcl-2-BH4 peptide whereas it was unaffected by the Bcl-2-BH4-IV/GG peptide.

To assess the IP_3_R-binding properties of BH4-Bcl-2-IV/GG, we performed GST-pull-down assays using the Bcl-2-BH4-IV/GG peptide and the domain 3 of IP _3_R1 (GST-Domain 3, GST + aa 923-1581 of IP _3_R1) fused to GST. Domain 3 corresponds to a tryptic subdomain of IP _3_R1 containing the previously identified binding site for Bcl-2 [17,40]. Using SDS-PAGE, we showed that the Bcl-2-BH4–peptide strongly interacted with GST-Domain 3 while Bcl-2-BH4 IV/GG lost most of its IP_3_R-binding properties (Figure 2A). We quantified the binding from three independent experiments and observed no differences between GST-Domain 3 and GST for the binding of Bcl-2-BH4 IV/GG (Figure 2B). Therefore, the residual Bcl-2-BH4-IV/GG peptide interaction with GST-Domain 3 was considered as non-specific. Next, we evaluated the effect of Bcl-2-BH4 and Bcl-2-BH4 IV/GG-peptides on IP _3_R1-channel activity by utilizing the nuclear-membrane patch-clamp technique [27,41]. Nuclei were isolated from DT40 cells stably expressing IP _3_R1 and channel openings were detected in the presence of submaximal doses of IP_3_ (1 µM) and of 5 mM ATP and 200 nM Ca^2+^. Figure 2C shows representative traces of IP _3_R1-channel openings at a pipette holding potential of -100 mV in the presence or absence of the different BH4 peptides. IP _3_R1-channel activity is decreased by the Bcl-2-BH4 peptide (50 µM), in agreement with earlier reports [17,32]. The same concentration of Bcl-2-BH4 IV/GG peptide in contrast had no effect (Figure 2C). Bcl-2-BH4 peptide reduced IP _3_R1-open probability (Po) by approximately 80% from 0.2 ± 0.02 to 0.04 ± 0.01, whereas Po values in the presence of the Bcl-2-BH4 IV/GG peptide were 0.24 ± 0.03 and didn’t significantly deviate from the control values (Figure 2D).

These data indicate that the IV/GG substitution not only destabilized the α-helix of Bcl-2-BH4 peptide but also abrogated its binding to the IP_3_R and its effect on IP_3_R-channel activity.

### In contrast to BH4-Bcl-2, BH4-Bcl-2 IV/GG does not inhibit IP_3_-induced Ca^2+^ release (IICR) in permeabilized and intact cells

**Figure 3:**
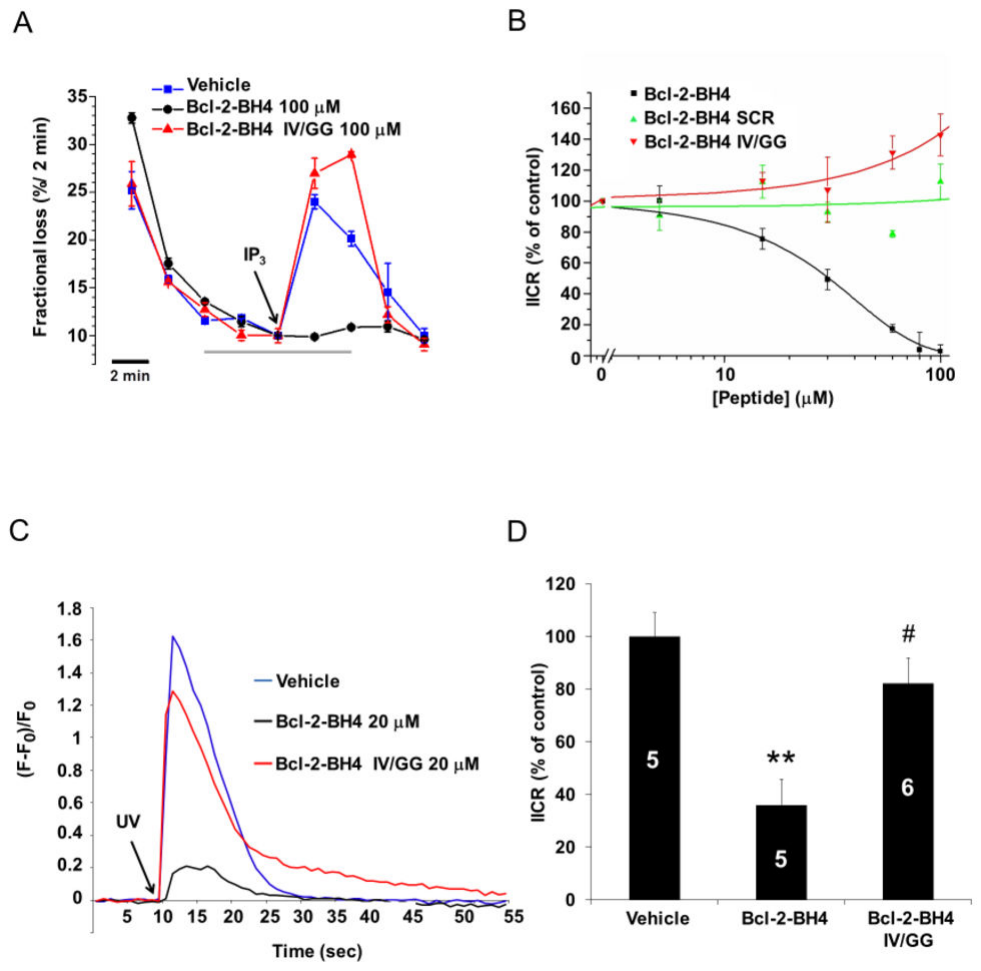
IV/GG substitution abrogates the inhibitory effect of Bcl-2-BH4 on IICR. (A) Representative unidirectional ^45^Ca^2+^ fluxes in permeabilized MEF cells plotted as fractional loss (% / 2 min) as a function of time. Ca^2+^ release was activated 10 min after starting the experiment by applying 3 µM IP_3_ (arrow) in the absence or presence of 100 µM of the different BH4-domain peptides (a gray bar indicates the peptide incubation period). (B) Concentration–response curves ([peptide] = 0,1; 3; 15; 30; 60; 100 µM) are shown for Bcl-2-BH4, Bcl-2-BH4 IV/GG and Bcl-2-BH4 SCR, obtained from 3 independent experiments. IICR was quantified as the difference of the fractional loss after 2 min of incubation with IP_3_ and the fractional loss before the IP_3_ addition. The 100% value corresponds to IICR in the presence of the vehicle and all the raw values were normalized to this control. Data points represent means ± SEM. (C) [Ca^2+^]_cyt_ increases in C6 glioma cells after photoliberation of caged IP_3_ at 9980 ms of recording (arrow). Traces of individual cells are displayed that were loaded with different BH4-domain peptides (20 μM) together with caged IP_3_ (50 μM). (D) Quantitative analysis of the area under the curve obtained from 5 or 6 independent experiments (as marked on each bar). Data were normalized to the vehicle condition, which was set as 100%, and are plotted as means ± SEM. These data indicate that Bcl-2-BH4 peptide significantly inhibited IICR (**), whereas Bcl-2-BH4 IV/GG peptide did not. # specifies the statistically significant difference between Bcl-2-BH4 and Bcl-2-BH4-IV/GG results.

Next, to verify whether these effects could be reproduced in cellular systems, we compared the regulation of IICR by the Bcl-2-BH4 domain and its IV/GG mutant using unidirectional ^45^Ca^2+^-flux assays in permeabilized MEF cells. This assay allows the quantitative assessment of Ca^2+^-efflux properties under unidirectional conditions in the absence of ER and mitochondrial Ca^2+^-uptake activity. The Ca^2+^ efflux of non-mitochondrial Ca^2+^ ***-***stores, loaded to steady state with ^45^Ca^2+^, is expressed as fractional loss (the amount of Ca^2+^ leaving the store in a 2-min time period divided by the total store Ca^2+^ content at that time). Adding IP_3_ (3 µM) to the efflux medium provoked IICR from the ER Ca^2+^ stores, which is observed as a peak increase in the fractional loss (Figure 3A). As hypothesized, the incubation with Bcl-2-BH4 (0.1, 3, 15, 30, 60, 100 µM) caused a potent concentration-dependent inhibition of IICR (IC_50_^≈^ 30 µM) while neither Bcl-2-BH4 IV/GG (Figure 3A-B) nor the scrambled version of the Bcl-2-BH4 peptide (Bcl-2-BH4 SCR) (Figure 3B) significantly affect IICR. In addition, we examined the effect of Bcl-2-BH4 or Bcl-2-BH4-IV/GG peptides on the IICR in intact C6 glioma cells. We loaded each peptide together with caged IP_3_ in a small and defined zone of an adherent culture using an *in situ* electroporation technique [34], and successively released IP_3_ by UV-flash photolysis in this area. While Bcl-2-BH4 inhibited the IICR, this was not the case for Bcl-2-BH4 IV/GG (Figure 3C-D). Both, the ^45^Ca^2+-^flux and the caged IP_3_-release assays independently indicated that the inhibition of IICR by the Bcl-2-BH4 peptide was abrogated upon destabilization of its α-helical structure.

### In contrast to BH4-Bcl-2, Bcl-2-BH4 IV/GG does not protect against staurosporine (STS)-induced apoptosis

**Figure 4:**
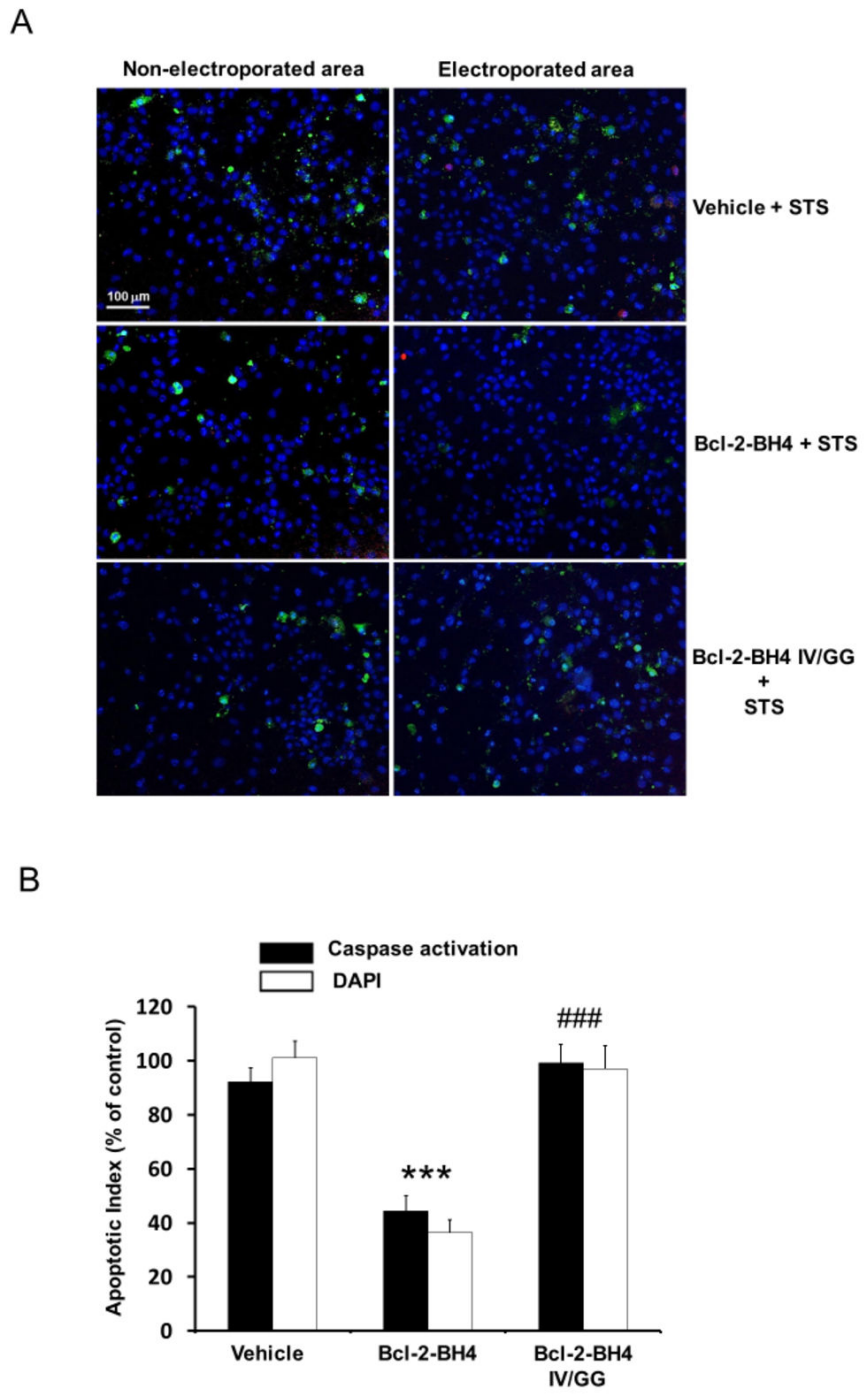
IV/GG substitution abolishes BH4-Bcl-2’s protective function against STS-induced apoptosis. Simultaneous analysis of caspase activation (FITC-VAD-FMK, green) and nuclear fragmentation (DAPI staining, blue fragments) of STS-treated C6 glioma cells. (A) Representative images of cells electroporated with or without BH4 peptides (20 µM) and successively treated with STS (2 µM for 6 h). The left images are taken outside the electroporation area and are used as negative controls (A, upper right). Electroporation in the absence of peptides (vehicle) (A, middle right). Electroporation of Bcl-2-BH4 peptide (A, lower right). Electroporation of Bcl-2-BH4-IV/GG peptides. Red color is due to the spillover into the FITC channel of the intense DTR signal (the electroporation loading control). (B) Quantitative image based AI (number of apoptotic cells divided by the total cell number). The AI was normalized to the AI outside the electroporated area. All results were obtained from 5 independent experiments and are plotted as means ± SEM. Only Bcl-2-BH4 loading significantly reduced the AI when compared with the control vehicle (**). ### indicates that the results obtained with Bcl-2-BH4 IV/GG were significantly different from Bcl-2-BH4.

Since STS is known to trigger Ca^2+^-dependent apoptosis [42,43], our next step was to compare the protective activity of Bcl-2-BH4 and Bcl-2-BH4 IV/GG in STS-treated C6 glioma cells. Two typical biochemical hallmarks of apoptosis are caspase-3 activation and downstream DNA fragmentation, detectable by specific fluorescent probes (here by FITC-VAD-FMK and DAPI, respectively). Therefore, we related both events to the magnitude of the ongoing apoptosis in C6 glioma cultures subsequent to the loading of Bcl-2-BH4 or Bcl-2-BH4 IV/GG (20 µM) and treatment for 6 h with STS (2 µM). The AI was calculated and expressed relative to the AI of the control condition outside the electroporated area. As shown in Figure 4, Bcl-2-BH4 peptide significantly reduced apoptotic cell death in STS-treated cells while Bcl-2-BH4 IV/GG behaved similarly to the vehicle. The latter demonstrated that the BH4-IV/GG peptide lost its ability to inhibit the IP_3_R and also its ability to counteract STS-induced apoptosis.

### Similarly to the IV/GG mutant, other glycine mutations that destabilize the α-helix of Bcl-2’s BH4 domain also abolish the IP_3_R-inhibitory properties

**Figure 5:**
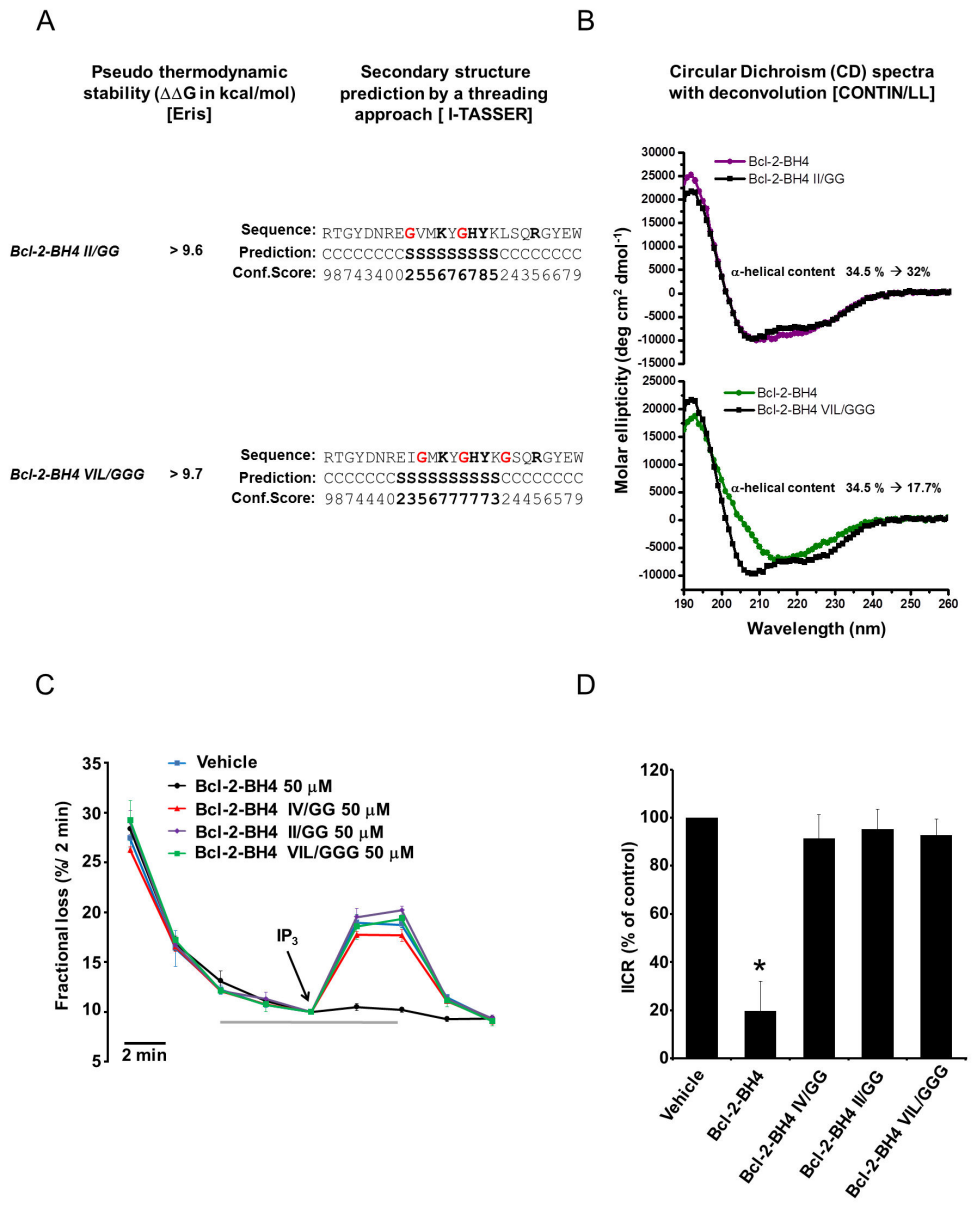
Other glycines substitutions that destabilize BH4-Bcl-2’s **α-helical structure abolish its IP_3_R-inhibitory properties**. (A, left) Panel showing the values of ΔΔG, in kcal/mol, resulting from the *in* *silico* analysis (Eris automated estimator) of the II/GG and VIL/GGG substitutions. Positive ΔΔG values indicate destabilizing mutations (A, right). Predicted-secondary structure assignments for the isolated BH4 domain mutated as described above. Each panel shows (from top to bottom) the primary structure, the secondary-structure predictions (C = random coil, S = strand [black/bold]) and the level of confidence of the predictions (confidence scores from 0 to 9). The key residues involved in the regulation of IP _3_Rs are depicted in black/bold while the position of the exchanged residues is indicated by the red G residues in the primary structure. (B) CD spectra of synthetic Bcl-2-BH4 (black line) in comparison with the ones for its G-substituted counterparts [II/GG (purple trace), VIL/GGG (green trace)]. The ellipticity is calculated per mole of amino-acid residue. Both mutant peptides showed a relative decrease in α-helical conformation as assessed by spectra analysis with the CONTIN/LL deconvolution method (see provided change in α-helical percentage for each condition. For the percentages of the other secondary structure features see Table S1 1). (C) Representative unidirectional ^45^Ca^2+^ fluxes in permeabilized MEF cells plotted as fractional loss (% / 2 min) as a function of time. Ca^2+^ release was activated 10 min after starting the experiment by applying 3 µM IP_3_ (arrow) in the absence or presence of 50 µM of the different BH4-domain peptides (the traces are color coded as in B). The gray bar indicates the peptide-incubation period. Data points represent means ± SD (D) IICR was quantified as the difference of the fractional loss after 2 min of incubation with IP_3_ and the fractional loss before the IP_3_ addition in the presence of vehicle (DMSO), Bcl-2-BH4 and the respective mutant peptides. The 100% value corresponds to IICR in the presence of the vehicle. All values were normalized to this control. Data points represent means ± SEM. * indicates a statistically significant difference from vehicle control.

To further examine the importance of the α-helical organization of the BH4 domain, we introduced other dicodon and tricodon mutations in the BH4 domain peptide. The selected amino acids likely resemble the backbone of the α-helix in the full-length protein and are not surface accessible in the full-length protein. Introducing glycine residues in the BH4 domain of Bcl-2 at positions I14/I19 (Bcl-2-BH4 II/GG), V 15/I19/L23 (Bcl-2-BH4 VIL/GGG), resulted in structure destabilization (positive ΔΔG values obtained *via* the Eris server, Figure 5A, left results) and predicted reduced α-helical organization via I-TASSER (Figure 5A, right results). This was validated by CD-spectroscopy, which confirmed the reduced α-helical content for the two mutant peptides (Figure 5B). While Bcl-2-BH4 VIL/GGG displayed a prominent decrease in α-helical content, Bcl-2-BH4 II/GG only displayed a minor but consistent decrease in α-helical content (for the percentages of the different secondary structure features see also Table S1). By using unidirectional ^45^Ca^2+^ flux assays in permeabilized MEF cells, both BH4-Bcl-2 mutant peptides lost their ability to inhibit IP_3_R-mediated Ca^2+^ release (wild-type BH4-Bcl-2 and Bcl-2 IV/GG were used as positive and negative controls, respectively) (Figure 5C).

## Discussion

Here, we examined the efficacy of Bcl-2-BH4 as an IP_3_R-inhibitory peptide in relation to its intrinsic secondary structure. Our findings indicate that the α-helicity of Bcl-2-BH4 peptide is a key determinant for its ability to directly suppress IP_3_R signaling and Ca^2+^-mediated apoptosis. Mutations that destabilize the α-helix and/or reduce the α-helical content of the Bcl-2-BH4 peptide abolished its IP _3_R1-inhibitory properties. Purposely, we have focused on targeting amino acids that are not surface accessible in the full-length protein but appear to be critical for the proper α-helical organization of the BH4 domain.

Irrespective of any concerns on structural organization or selectivity, many studies already showed that a cell-penetrating peptide comprising the sequence for Bcl-2’s BH4 domain is protective against various cellular stressors [44–46]. Such activity can be attributed to a large extent to the direct inhibition of the IP _3_Rs by Bcl-2’s BH4 peptide as reported in our recent studies [12,17,32]. This may not be surprising given that in the native protein the Bcl-2-BH4 domain adopts an α-helical conformation, a secondary structure motif involved in many protein-protein interactions as well as in protein association with cellular membranes [47,48]. Yet, it is important to note that synthetic peptides derived from native protein sequences might shift conformations between helices, sheets and random coils, since they have been removed from the stabilizing effects of their protein context.

Isolated peptides corresponding to the BH4 domain of Bcl-2 have equally been reported to adopt different secondary structures according to the surrounding environment: from an α-helix or a β-sheet in an amphipathic/membrane-like environment to a random coil when present in an aqueous medium [22,49,50]. Here, we show that the Bcl-2’s BH4 peptide has a substantial α-helical content (Figure 1C), correlating with its ability to suppress the activity of the IP _3_Rs and protect against STS-induced apoptosis. Consequently, introducing a double glycine amino acid in its α-helical core not only disrupted the characteristic secondary structure of the peptide, but also abrogated its ability to bind to the IP _3_R1 and reduced the related pro-apoptotic Ca^2+^ signaling. From our CD analysis, we estimated that approximately 35% of the Bcl-2’s BH4 peptide is organized in an α-helix when solubilized in the membrane-mimicking agent TFE [51]. The latter data correlate well with previous reported values, for example the 27% obtained by Lee et al. [22] in detergent micelles and the 45% obtained by Khemtemourian et al. [49] in TFE. The apparent discrepancy between the various studies can be explained by the slightly different experimental conditions (pH, temperature, solvent purity, membrane environment and peptide length), which can drastically affect the peptide’s secondary structure. However, it is clear from our data and from the previous work that this peptide displays a stable α-helical conformation in a membrane-mimicking environment [22,49]. A membrane- or detergent-enriched solution resembles the most the physiological or experimental conditions in which the IP_3_R/Bcl-2 interaction presumably occurs, since they are both membrane proteins. Therefore, the BH4 region of the small Bcl-2 protein would be located in the proteinaceous binding pocket on the IP_3_R, in the vicinity of the membrane environment This is compatible with the relatively high doses of BH4 peptide necessary in the electrophysiological assays (Figure 2C-D) and in the ^45^Ca^2+^ flux assays (IC_50_ ≈ 30 µM, Figure 3B), which is likely due to the mixed α-helix/unstructured/β-sheet conformations present in the largely aqueous medium [22,49,52]. Accordingly, Bcl-2’s BH4 peptide, which is amphiphilic and has a net positive charge at physiological pH, would still need to accumulate onto the acidic membrane environment of the ER and to assume a more α-helical conformation for gaining its ability to inhibit the IP _3_Rs. Conversely, the IV/GG-mutated BH4-peptide shows a decrease of approximately 50% in the extent of helix content (34.5% *vs* 18.9%), indicating a collapse of the α-helix in Bcl-2-BH4 IV/GG. The experimental evidence from the CD spectra suggests a shift in the secondary structure of the peptide, which may cause a mispositioning of the key BH4 amino acids necessary for its specific IP_3_R inhibition and consequent protective role. This is supported by the fact that other α-helical-destabilizing amino acid substitutions in the “backbone” of the BH4 domain of Bcl-2 result in a complete loss of IP_3_R-inhibitory properties. Remarkably, not only alterations that cause drastic changes in the α-helical content (like in Bcl-2-BH4 VIL/GGG) but also alterations that only trigger minor changes in the α-helical content abolish (like in Bcl-2-BH4 II/GG) the IP_3_R-inhibitory properties of the BH4 domain of Bcl-2. These data indicate that the proper structural organization of the BH4 domain of Bcl-2 is critical for its functional properties, and a slight deviation of this structure may abolish its inhibitory effect on the IP_3_R. These data are in line with our previous observations, indicating that multiple amino acids in the BH4 domain of Bcl-2 contribute to binding and inhibiting IP _3_Rs [12]. A slight distortion of some of these amino acids in the BH4 domain of Bcl-2 likely will be sufficient to abolish the IP_3_R-inhibitory properties of this peptide.

Hence, our results indicate that a precise secondary structure of the Bcl-2-BH4 peptide determines its specific dampening of the IP_3_R-dependent Ca^2+^ signaling and the resulting protection against Ca^2+^-mediated apoptosis. Nonetheless, further structure-activity relationship studies are still necessary to interpret our findings in the context of the full-length Bcl-2 interaction with the IP _3_Rs and in relation to the unknown mechanism of IP_3_R channel inhibition [53]. Another challenge is to obtain from this anti-apoptotic peptide a stable, cell-permeable IP_3_R-inhibitory tool that retains the specificity of the native BH4-domain α-helix. Notably, the recent successes of two α-helix-stabilizing techniques, like the all-hydrocarbon and the triazole stapling [54,55] have already paved the way for accomplishing this task.

In conclusion, our study provides new opportunities for the rational design of selective IP_3_R-inhibitors. Furthermore, it lays the basis for the development of a novel class of Bcl-2-BH4-derived molecules targeting disorders associated with aberrant intracellular Ca^2+^ signaling.

## Supporting Information

Table S1 CD traces have been deconvoluted by using the CONTINLL algorithm of the CDPro software. The measured CD spectrum is compared with the spectra of a set of reference peptides for which high quality X-ray diffraction data are available. The root mean square deviation (RMSD) between the experimental and the calculated CD spectra is an indication of the quality of the deconvolution. Lower values of RMSD indicate higher accuracy of the deconvolution. The obtained RMSD values are around the acceptable value of 0.1 [31,39].
(TIF)
