# Supplementary figures and images for: Alpha-Helical Destabilization of the Bcl-2-BH4-Domain Peptide Abolishes Its Ability to Inhibit the IP3 Receptor
Source: PLoS One. 2013 Aug 30;8(8):e73386. doi: 10.1371/journal.pone.0073386 (PMC3795776; doi:10.1371/journal.pone.0073386)

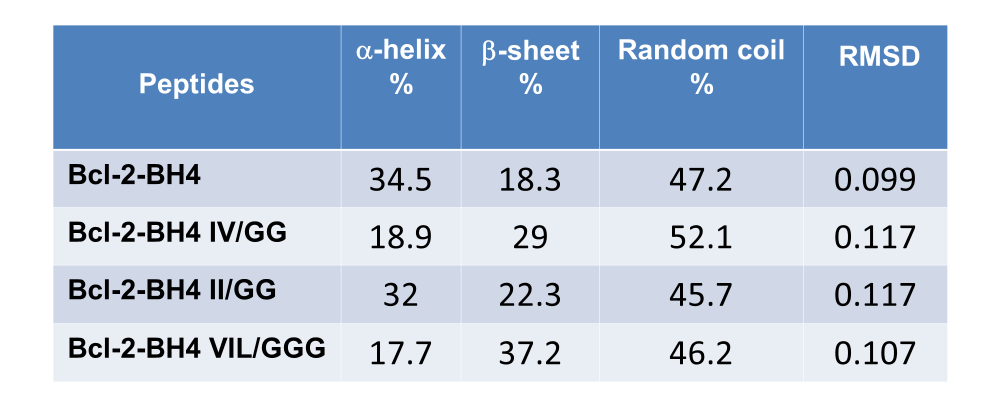

Supplement: Table S1 — CD traces have been deconvoluted by using the CONTINLL algorithm of the CDPro software. The measured CD spectrum is compared with the spectra of a set of reference peptides for which high quality X-ray diffraction data are available. The root mean square deviation (RMSD) between the experimental and the calculated CD spectra is an indication of the quality of the deconvolution. Lower values of RMSD indicate higher accuracy of the deconvolution. The obtained RMSD values are around the acceptable value of 0.1 [31,39]. (TIF) [file pone.0073386.s001.tif]
